# Engaging Mortality: Effective Implementation of Dignity Therapy

**DOI:** 10.1089/jpm.2023.0336

**Published:** 2024-01-30

**Authors:** Diana J. Wilkie, George Fitchett, Yingwei Yao, Tasha Schoppee, Marvin O. Delgado Guay, Joshua Hauser, Sheri Kittelson, Sean O'Mahony, Michael Rabow, Tammie Quest, Sheldon Solomon, George Handzo, Harvey Max Chochinov, Linda L. Emanuel

**Affiliations:** ^1^ Department of Biobehavioral Nursing Science, College of Nursing, University of Florida, Gainesville, Florida, USA.; ^2^ Department of Religion, Health and Human Values, Rush University Medical Center, Chicago, Illinois, USA.; ^3^ Community Hospice & Palliative Care, Jacksonville, Florida, USA.; ^4^ Department of Palliative, Rehabilitation, and Integrative Medicine, MD Anderson Cancer Institute, Houston, Texas, USA.; ^5^ Department of Medicine, Northwestern University and Jesse Brown VA Medical Center, Chicago, Illinois, USA.; ^6^ Department of Medicine, University of Florida, Gainesville, Florida, USA.; ^7^ Department of Medicine, Rush University, Chicago, Illinois, USA.; ^8^ Department of Medicine, University of California San Francisco, San Francisco, California, USA.; ^9^ Department of Family and Preventive Medicine, Emory University, Atlanta, Georgia, USA.; ^10^ Department of Psychology, Skidmore College, Saratoga Springs, New York, USA.; ^11^ HealthCare Chaplaincy Network, New York, New York, USA.; ^12^ Department of Psychiatry and Cancer Care Manitoba Research Institute, University of Manitoba, Winnipeg, Canada.; ^13^ Mongan Institute, Harvard University, Boston, Massachusetts, USA; Supportive Oncology, Robert H Lurie Comprehensive Cancer Center, Northwestern Medical Group, Feinberg School of Medicine, Northwestern University Chicago, Illinois, USA.

**Keywords:** cancer, dignity impact, Dignity Therapy, older adults, outpatient, palliative care

## Abstract

**Background:**

Patients consider the life review intervention, Dignity Therapy (DT), beneficial to themselves and their families. However, DT has inconsistent effects on symptoms and lacks evidence of effects on spiritual/existential outcomes.

**Objective:**

To compare usual outpatient palliative care and chaplain-led or nurse-led DT for effects on a quality-of-life outcome, dignity impact.

**Design/Setting/Subjects:**

In a stepped-wedge trial, six sites in the United States transitioned from usual care to either chaplain-led or nurse-led DT in a random order. Of 638 eligible cancer patients (age ≥55 years), 579 (59% female, mean age 66.4 ± 7.4 years, 78% White, 61% stage 4 cancer) provided data for analysis.

**Methods:**

Over six weeks, patients completed pretest/posttest measures, including the Dignity Impact Scale (DIS, ranges 7–35, low-high impact) and engaged in DT+usual care or usual care. They completed procedures in person (steps 1–3) or via Zoom (step 4 during pandemic). We used multiple imputation and regression analysis adjusting for pretest DIS, study site, and step.

**Results:**

At pretest, mean DIS scores were 24.3 ± 4.3 and 25.9 ± 4.3 for the DT (*n* = 317) and usual care (*n* = 262) groups, respectively. Adjusting for pretest DIS scores, site, and step, the chaplain-led (*β* = 1.7, *p* = 0.02) and nurse-led (*β* = 2.1, *p* = 0.005) groups reported significantly higher posttest DIS scores than usual care. Adjusting for age, sex, race, education, and income, the effect on DIS scores remained significant for both DT groups.

**Conclusion:**

Whether led by chaplains or nurses, DT improved dignity for outpatient palliative care patients with cancer. This rigorous trial of DT is a milestone in palliative care and spiritual health services research.

clinicaltrials.gov: NCT03209440.

## Introduction

Research makes clear the importance of addressing central spiritual/existential issues and tasks among seriously ill patients.^1^ Dignity Therapy (DT) is a life review intervention resulting in a legacy document; it has inconsistent effects on a variety of symptom-related outcomes despite patients considering DT to be beneficial to themselves and their families.^2^ DT's focus on meaning making could facilitate preparation for death, life-completion tasks, awareness of cancer prognosis, and heightened will-to-live,^3^ especially for older adults. We studied DT's effects on spiritual/existential outcomes central to dignity when administered in real-life care settings, testing the efficacy of DT on dignity impact in a pragmatic multisite trial among older adults receiving outpatient palliative care.

In 441 terminally ill patients, 46% had impaired dignity with 7% reporting its severity as moderate or greater and having significantly greater desire for death than those whose dignity was intact.^4^ DT has been studied and implemented in countries around the world, has cross cultural resonance, and is easily adapted to accommodate cultural variation.^5^

We compared usual care, chaplain-led, and nurse-led DT for effects on dignity impact,^6^ existential tasks (death preparation, life completion),^7^ and cancer prognosis awareness (peaceful awareness of cancer prognosis, treatment preferences consistent with their prognosis).^8,9^ We hypothesized that DT groups would have higher scores on the dignity and existential tasks than the usual care group, and that more patients in the DT groups would report being peacefully aware with treatment preferences aligned to cancer prognosis than the usual care group.

## Methods

### Design

**FIG. 1.**
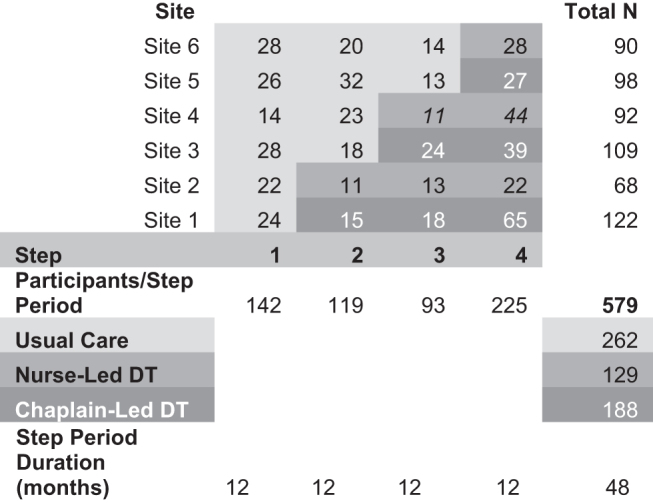
Actual study accrual by site and step for the step-wedge design. DT, Dignity Therapy.

In a pre/posttest randomized clinical trial (RCT) with a 4-step (∼12 months per step), stepped-wedge design,^10^ we randomized half of the six sites to chaplain-led DT and half to nurse-led DT (Fig. 1). All sites were assigned to the control condition (usual outpatient palliative care) in the first step and at each successive step, one chaplain-led site and one nurse-led site transitioned to the intervention (usual outpatient palliative care+DT). The stepping-up order among chaplain-led sites and nurse-led sites was randomized separately using (uniformly distributed) random integer (1–6, corresponding to six possible ways of ordering three sites) generators. Therefore, all sites were in the intervention during the last step, which occurred during the COVID-19 pandemic.

A stepped-wedge design was chosen since in the clinical environment it would be difficult for chaplains and nurses, once they had received extensive DT protocol training, to withhold DT from patients individually randomized to usual care. The Institutional Review Board at each site approved the study (clinicaltrials.gov: NCT03209440).

### Setting

Palliative care specialist-directed outpatient palliative care programs at four National Cancer Institute designated cancer centers and two academic cancer centers across the United States provided access to patients and resources for in-person or virtual interactions during steps 1–3. During step 4, all interactions, including the DT intervention, occurred virtually.

### Sample

The recruited sample met *inclusion criteria*: diagnosed with cancer, receiving outpatient palliative care, age ≥55 years, able to speak/read English, and physically able to complete the study (Palliative Performance Scale [PPS] >50,^11–13^ suggesting a mean life expectancy of at least 55 days at the time of enrollment).^13^ *Exclusion criteria* were the following: legally blind, cognitively unable to complete study measures (Mini Mental Status Exam <24^14^: steps 1–3; unable to spell “world” backwards: step 4), medical record diagnosis of psychosis, or enrolled in another intervention study that focused on similar concepts.

From the DT treatment effect estimated in prior research,^6^ we calculated *a priori* power for two-sided significance level (0.025) under different levels of site disparity as indexed by the intraclass correlation (ICC).^15^ With minimal site disparity (ICC = 0), a sample of 560 had 95% power to detect the DT treatment effect; with more severe site disparity (ICC = 0.4), the power would lower to 80%.

### Procedure

**FIG. 2.**
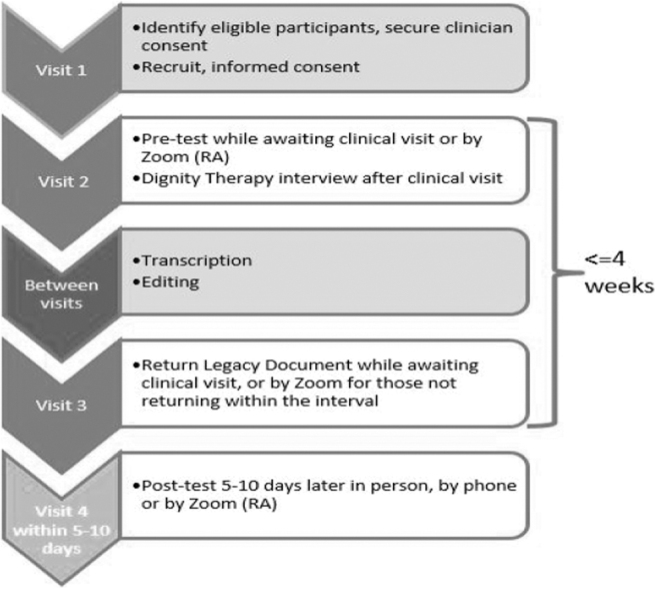
Flow of patients through the study procedures.

As per the protocol article,^16^ Figure 2 shows the general flow of patients through study procedures over four to six weeks. Research assistants (RAs) were trained in all study procedures. A clinic team member referred patients to the RAs at palliative care clinic visits or by phone after a virtual visit. RAs recruited patients and obtained written consent. For those eligible, the RAs scheduled a convenient time for the patient to complete the pretest, with the RAs reading the questions to the patient and recording the responses in a REDCap database. Since blinding of the RAs was not possible in the stepped-wedge design, the data collection process was audio recorded to allow for validation of patient responses and minimize bias.

**FIG. 3.**
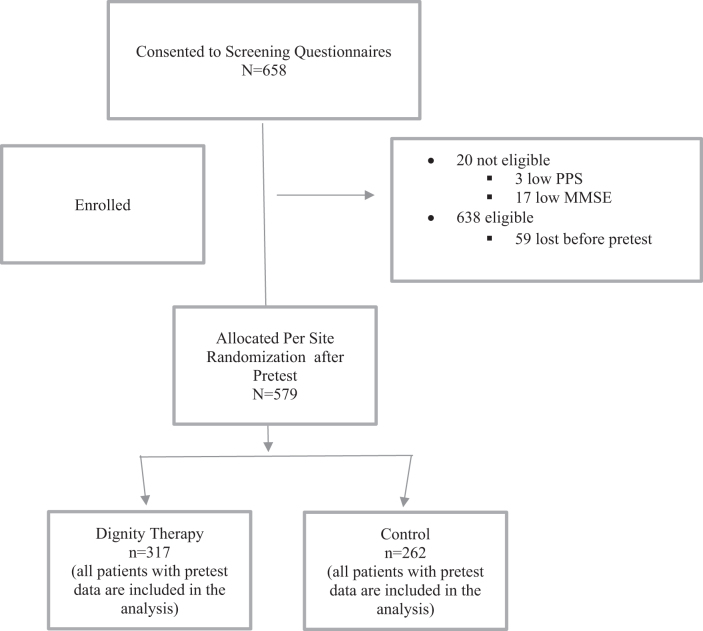
CONSORT diagram of study patients screened, enrolled, allocated to treatment groups, and analyzed. MMSE, Mini Mental Status Exam; PPS, Palliative Performance Scale.

Usual care or DT was delivered over the next three to four weeks, either in-person if the patient had a clinic visit or by telephone contact. RAs scheduled follow-up appointments and completed the posttest measures within 5–10 days of the delivery of the legacy document or the end of the 4-week period for the usual care group (Fig. 3). Upon completion of posttest measures, the patient received a $50 cash/gift card. During step 4, procedures were conducted virtually via telephone or Zoom for safety during the COVID-19 pandemic.

### Intervention

Nurses (registered nurses or advanced practice nurses and certified in palliative care) or chaplains (Board-Certified Chaplains and associated with the palliative care clinics but not necessarily specifically trained in palliative care) provided the intervention.

#### Usual care

The specialty-based palliative care team providing clinic-based outpatient palliative care were palliative care board-certified physicians and nurse practitioners and supported by clinic nurses. The clinics included chaplain support for patients only as needed from the medical center's chaplaincy staff.

#### Dignity Therapy

The DT intervention followed a manualized guide (Supplementary Table S1).^17^ The chaplain-led or nurse-led DT intervention involved four patient contacts that followed a set process that facilitated a personal process of reflection and recognition allowing the patient to make meaning of their experience (Supplementary Tables S2 and S3). As described elsewhere,^17,18^ the DT creator (Chochinov) provided a two-day, standardized training for study chaplains and nurses immediately before a study site stepped up to DT, posttraining fidelity-driven activities included a standardized-patient process, tailored-to-performance feedback, and peer support for therapists.^18^

As described elsewhere,^19^ we monitored intervention fidelity with a modified tool.^17^ Trained RAs read each original DT transcript and used the fidelity tool to document the nurse's/chaplain's protocol adherence. Possible scores ranged from 0 to 15. For 93% of the 219 transcripts monitored, the fidelity score was ≥12 indicating that the 80% criterion for fidelity was met as an indication of rigor and reproducibility.

Professional transcriptionists provided verified transcripts of the typically 60-minute DT interviews. From the transcripts, two DT protocol-trained editors created the legacy documents.

### Measures

We used standard, valid, and reliable measures to screen patients for study eligibility: Palliative Performance Scale,^11–13^ Mini Mental Status Examination,^14^ Edmonton Symptom Assessment Scale Revised,^20^ and 14-item Religious and Spiritual Struggles Scale.^21^

#### Dignity impact

Our primary outcome measure was the 7-item *Dignity Impact Scale* (DIS, Supplementary Table S4 shows items).^6^ The responses are scaled from 1 (strongly disagree) to 5 (strongly agree). The DIS score is the sum of item scores; can range from 7 to 35 with higher scores representing better dignity impact. Items have been used in many DT studies with evidence of their face validity in the target population.^4,22,23^ Content validity of the DIS is supported by the well accepted conceptual domains of dignity.^4^ Validity of the DIS is also supported by factor analysis of the current sample indicating that a one factor model fits the data with a Root Mean Square Error of Approximation Index (0.07) and a Tucker-Lewis Index (0.94). Cronbach's *α* was 0.85 in a previous sample, which indicated high reliability. In our sample, the Cronbach's *α* was 0.80 (baseline) and 0.83 (posttest). The DIS was sensitive to intervention effects in a large RCT of DT using posttest data in a secondary analysis.^6^

#### Existential tasks

The existential tasks were measured with the valid and reliable *preparation for death* and *life completion* subscales of the QUAL-E.^7^

#### Cancer prognosis awareness

We obtained data from two measures related to cancer prognosis awareness: peaceful awareness and treatment.^8,9^

We measured *peaceful awareness* with the two items.^8,9^ *Terminal illness awareness* focused on terminal illness acknowledgment, in which patients rated their current health status as “relatively healthy,” “seriously but not terminally ill,” or “seriously and terminally ill.” *Peacefulness* focused on the frequency of feeling deep inner peace or harmony, which was rated on a 6-point Likert scale ranging from “never or almost never” to “many times a day.” Scores of at least three on each of the two items defined positive peaceful awareness, a dichotomous measure.

We measured *treatment preferences* with a single item from a standardized and validated measure.^8^ Two options, “prolong life; treat everything” and “attempt to cure, but reevaluate often,” were considered as “life prolonging” treatment choices. The other two options, “limit to less invasive and less burdensome intervention” and “provide comfort care only,” were considered “nonlife prolonging” treatment choices. The response options were treated as an ordinal scale with higher scores representing choices for nonlife prolonging treatments.

### Analysis

We used statistical software R and intent-to-treat analysis of all patients with pretest data included in the analysis. For missing data processing, we utilized multiple imputation to generate multiple completed datasets upon which inferences were performed and then aggregated. The amount of missing data was small at pretest (2%) and at posttest (2%) for patients who completed the posttest. The main source of missing data for patients not completing the posttest (22%) was attrition due to declining health or death.

We obtained descriptive statistics for demographic variables and outcome variables and utilized mixed-effect regression models with random effect terms to account for between-site differences. We also included steps in our model to account for potential change over time. To estimate the effects of DT on an outcome variable at posttest, we adjusted for the pretest value of the corresponding outcome. Significance was set *a priori* at a two-sided alpha of 0.025 for effects of chaplain-led and nurse-led DT and 0.05 otherwise.

## Results

### Sample characteristics

Of the 658 consented patients, 20 were not eligible (Fig. 3). There was attrition of 59 before pretest resulting in an enrolled sample of 579 patients between August 2017 and October 2021. Of these 579 patients, 317 were in the DT group and 262 in the usual care group (Fig. 2).

The mean age of the sample was 66.4 ± 7.4 years (Table 1). The sample was 59% female, 60% married or partnered, 82% having at least some college, 87% reporting a religion, and 54% having metastatic cancer. The sample was majority White (*n* = 448) or Black (*n* = 103) (Table 1). We consolidated the race into two categories: White and Other.

**Table 1.** Sample Demographic Characteristics (*N* = 579)

Variable (% missing)	Category	All	Usual care	DT	*p*
Age (0%)	Mean ± SD	66.4 ± 7.4	67.2 ± 7.5	65.7 ± 7.3	0.03
Sex (0%)	Female	59%	56%	62%	0.05
	Male	41%	44%	38%	
Race (1.2%)	Other^a^	22%	29%	15%	0.01
	White	78%	71%	85%	
Ethnicity (2.6%)	Hispanic or Latino	6%	5%	7%	0.39
	Not Hispanic or Latino	94%	95%	93%	
Marital status (7.9%)	Married/partnered	60%	61%	59%	0.73
	Single	40%	39%	41%	
Education level (11.1%)	High school or less	18%	24%	13%	0.004
	Some college	32%	30%	33%	
	College	25%	23%	27%	
	Advanced degree	26%	23%	28%	
Family annual income (16.6%)	Less than $10,000	8%	8%	7%	0.08
	$10,000 to $19,999	12%	12%	11%	
	$20,000 to $29,999	10%	11%	9%	
	$30,000 to $39,999	8%	8%	9%	
	$40,000 to $49,999	7%	6%	7%	
	$50,000 or more	56%	55%	57%	
Christian (8.8%)	No	24%	19%	28%	0.13
	Yes	76%	81%	72%	
Have a religion (8.8%)	No	13%	12%	15%	0.67
	Yes	87%	88%	85%	
Cancer stage (6.4%)	1	12%	10%	14%	0.24
	2	7%	7%	7%	
	3	20%	22%	19%	
	4	61%	61%	60%	
Time since: diagnosis (years) (2.1%)	Mean ± SD	3.8 ± 4.6	4.3 ± 4.9	3.4 ± 4.2	0.06
Metastasized (16.9%)	Yes	54%	56%	53%	0.40
Palliative Performance Score (0%)	60	18%	15%	20%	0.19
	70	32%	32%	32%	
	80	30%	31%	30%	
	90	16%	19%	14%	
	100	4%	4%	4%	

^a^ Other race included 131 patients who report race as follows: 103 Black, 5 Asian, 2 Pacific Islander, 1 Native American, 13 other races, and 7 unknown race.

DT, dignity therapy; SD, standard deviation.

Only 18% of the patients had the minimum eligible PPS score of 60; the vast majority had PPS scores between 70 and 100 (Table 1). PPS score difference between the DT and usual care groups was not significant (*p* = 0.19).

On average, the sample reported generally low physical-emotional distress and medium spiritual distress (Table 2). The pretest mean physical-emotional distress was significantly higher for the DT group than the usual care group (43.9 ± 13.0 vs. 40.9 ± 13.2; *p* = 0.02). Similarly, the pretest mean spiritual distress was significantly higher for the DT group than the usual care group (4.4 ± 5.2 vs. 3.2 ± 4.6; *p* = 0.05).

**Table 2.** Pretest Values for Distress Levels and Patient Outcomes (*N* = 579)

Variable (possible range of scores) [% missing]	Category	All	Usual care	DT	*p*
Physical distress (0–90) [0.3%]	Mean ± SD	42.6 ± 13.1	40.9 ± 13.2	43.9 ± 13.0	0.02
Spiritual distress (0–56) [0.8%]	Mean ± SD	3.8 ± 4.9	3.2 ± 4.6	4.4 ± 5.2	0.05
Dignity Impact Score (7–35) [2.6%]	Mean ± SD	25.0 ± 4.4	25.9 ± 4.3	24.3 ± 4.3	0.001
Preparation (4–20) [1.9%]	Mean ± SD	15.0 ± 3.4	15.4 ± 3.2	14.7 ± 3.5	0.02
Completion (7–35) [2.1%]	Mean ± SD	27.0 ± 5.6	26.8 ± 5.2	27.3 ± 5.8	0.44
Terminal illness awareness (1–3) [2.2%]	1 = Relatively healthy	39%	41%	38%	0.93
	2 = Seriously but not terminally ill	34%	34%	35%	
	3 = Seriously and terminally ill	26%	26%	27%	
Peacefulness (1–6) [.6%]	1 = Never or almost never	3%	2%	3%	0.20
	2 = Occasionally	12%	12%	13%	
	3 = Some days	18%	17%	19%	
	4 = Most days	34%	31%	36%	
	5 = Every day	16%	18%	14%	
	6 = Many times a day	17%	20%	15%	
Peaceful awareness [2.1%]	Peacefully aware	23%	23%	23%	0.42
	Not peacefully aware	77%	77%	77%	
Treatment preference (1–4) [3.3%]	Prolong life; treat everything	12%	14%	11%	0.02
	Attempt to cure, but reevaluate often	31%	34%	28%	
	Limit to less invasive and less burdensome interventions	21%	21%	21%	
	Provide comfort care only	36%	31%	40%	

### Primary outcome: DIS

At pretest, the mean DIS score was 24.3 ± 4.3 in the DT group and 25.9 ± 4.3 in the usual care group. The mean posttest DIS scores for the DT group and the usual care group were 26.2 ± 4.1 and 26.3 ± 4.7, respectively. Adjusting for pretest DIS scores, study step, and site, the DT group reported significantly higher posttest DIS scores (*β* = 1.8, *p* = 0.005) than the usual care group (Table 3), corresponding to an effect size of 0.4. The interaction between race and DT was not significant (*p* = 0.73). The size of DT effects was similar for White (*β* = 1.9, *p* = 0.006) and the Other race (*β* = 1.6, *p* = 0.06) patients.

**Table 3.** Estimates of Treatment Effects for Primary and Secondary Outcomes

Outcome	Estimate	Standard error	*t*	*p*
Primary				
Dignity impact	1.849	0.661	2.800	**0.005**
Secondary				
Preparation	−0.697	0.442	−1.578	0.12
Completion	0.887	0.638	1.390	0.17
Peaceful awareness	0.976	0.564	1.732	0.08
Treatment preferences	0.252	0.159	1.584	0.11

Bold indicates *a priori* significance level met.

Other covariates included in the regression model were pretest values of the corresponding outcome, study step, and study site.

The chaplain-led (*β* = 1.7, *p* = 0.023) and nurse-led (*β* = 2.1, *p* = 0.005) DT groups both reported significantly higher posttest DIS scores than the usual care groups (Table 4). Adjusting for age, sex, race, education, and income, the effect on DIS scores remained significant for both DT groups. The two groups did not differ significantly on posttest DIS scores (*p* = 0.48).

**Table 4.** Estimates of Treatment Effects for Primary and Secondary Outcomes by Chaplain-Led and Nurse-Led Dignity Therapy

Outcome	Predictor	Estimate	Standard error	*t*	*p*
Primary					
Dignity impact	Chaplain-led DT	1.671	0.731	2.285	**0.023**
	Nurse-led DT	2.068	0.741	2.793	**0.005**
Secondary					
Preparation	Chaplain-led DT	−0.617	0.486	−1.270	0.21
	Nurse-led DT	−0.790	0.503	−1.572	0.12
Completion	Chaplain-led DT	0.901	0.724	1.244	0.21
	Nurse-led DT	0.912	0.731	1.248	0.21
Peaceful awareness	Chaplain-led DT	1.000	0.618	1.619	0.11
	Nurse-led DT	0.955	0.637	1.499	0.14
Treatment preferences	Chaplain-led DT	0.345	0.174	1.982	**0.05**
	Nurse-led DT	0.134	0.178	0.748	0.46

Bold indicates *a priori* significance level met.

Other covariates included in the regression model were pretest values of the corresponding outcome, study step, and study site.

### Secondary outcomes

At pretest, the mean preparation score was 14.7 ± 3.5 in the DT group and 15.4 ± 3.2 in the usual care group. The mean posttest preparation scores for the DT group and the care group were 14.8 ± 3.2 and 15.6 ± 3.2, respectively. Adjusting for pretest preparation scores, study step, and site, DT group did not report significantly different posttest preparation scores than the usual care (*p* = 0.12).

At pretest, the mean completion score was 27.3 ± 5.8 in the DT group and 26.8 ± 5.2 in the usual care group. The mean posttest completion scores for the DT group and the care group were 27.8 ± 4.8 and 27.0 ± 4.9, respectively. Adjusting for pretest life completion, study step, and site, the DT group did not report significantly higher posttest life completion scores than the usual care group (*p* = 0.17).

The percentages of peacefully aware patients at pretest were 77% for both DT and usual care groups (Table 2). Adjusting for pretest, study step, and site, the DT group did not report significantly higher posttest peaceful awareness than the usual care (*p* = 0.08).

The sample treatment preference at pretest is shown in Table 2 for pretest. Adjusting for pretest treatment preference and study step, the DT group did not report more preference for not prolonging life goals of care than the usual care (*p* = 0.11). Further analysis revealed that, at posttest, the chaplain-led (*p* < 0.05) group but not the nurse-led (*p* = 0.46) group reported more treatment preference for not prolonging life goals of care than the respective usual care group (Table 4).

## Discussion

We are the first to test the proximal effects of DT on dignity in a large, multisite trial in older adults with cancer receiving outpatient palliative care. In this stepped-wedge, multisite RCT conducted, in part, during the COVID-19 pandemic, we found that whether led by DT-trained chaplains or nurses, DT was effective in improving dignity for older adults. The DT effects were retained when we adjusted for demographic characteristics, including sex. Importantly for health equity, the DT effects were similar for groups who were White or Other race, a smaller group that was predominately Black American. DT led by chaplains but not by nurses was associated with more preference for goals of care focused on not prolonging life. In contrast, within the context of outpatient palliative care, DT had limited effect on other secondary outcomes focused on preparation for death, life completion, and peaceful awareness of terminal illness.

A central hypothesis of our study was that DT influences important spiritual and existential domains, such as the need for meaning, concerns about family, and need for a legacy,^4^ which are included in our primary efficacy outcome, the DIS. Although DT has been studied widely with promising benefit in many small RCTs and one large multinational RCT,^4,22–27^ ours is the first large-scale RCT to demonstrate efficacy on the primary outcome. We did so in real-life palliative care settings as older adults lived with the struggles posed by cancer and many also adjusted to the challenges of the COVID-19 pandemic.

The assault on human worth and dignity during the pandemic may have influenced the baseline DIS of study groups. It was during the last study step, at the height of the pandemic, when all sites were assigned to the DT group. We observed lower DIS at baseline in the DT group, an unexpected finding. Nonetheless, the DT group significantly outperformed the usual care group at posttest adjusting for the baseline DIS score. The success of DT delivered virtually, another adjustment that was necessary during the pandemic, is an important finding with implications for clinical implementation.

To address the imbalances that we observed between the usual care and the DT steps within sites, namely change over time, we used three statistical approaches. First, we adjusted for the baseline DIS in our regression analysis of treatment effect. Second, we also included site itself as a control covariate in our models. Third, we also ran models, including other covariates that showed imbalance between groups (e.g., age, sex, race, education, income) and found that the treatment estimates remained substantively the same.

Reasons for the lack of DT consistent effect on the secondary outcomes are unclear. Since palliative care board-certified physicians provided care at all six sites, other effects of DT may have been minimized above that of usual palliative care on quality-of-life (QoL) outcomes. In a RCT with patients with advanced illness, Steinhauser found similar mean scores as ours on the preparation and completion measures, which were not responsive to an intervention specifically focused on life completion and preparation.^28^ The responsiveness of QoL measures has been an issue that requires additional research, especially in palliative care populations.^29^ Our trial findings on mental health outcomes are under review. Clinical trials of DT, however, have consistently indicated high degrees of patient satisfaction, suggesting that they may be experiencing benefits beyond our current capacity for measurement.

A few limitations warrant consideration. Attrition is common in palliative care studies of patients with cancer. Our loss to follow-up of 22%, although within our projected 20% to 30% attrition rate, may have unknown effects on external validity of findings. Our sample was intentionally focused on older adults with cancer; findings may not generalize to a younger population with cancer or other life-threatening conditions. Findings may not be reproducible with nurses or chaplains not sufficiently trained or experienced in palliative care. Our sample overrepresented the population with at least some college education and did not adequately represent the male or racial and ethnic minority populations. Although there is some evidence of the DIs' validity and reliability from a previous study,^6^ further research is warranted to improve understanding of its properties, including the minimal clinically meaningful difference.

In conclusion, DT led by either chaplains or nurses was effective in improving dignity for older adults with cancer also receiving outpatient palliative care. Our findings across racial groups also suggest that DT, a patient-centered approach, has a promise as an intervention to improve health equity in support of dignity for racial minorities with cancer. This rigorous trial of DT is a landmark milestone in oncology, palliative care, and spiritual health services focused research.

## Supplementary Material

Supplemental data

Supplemental data

Supplemental data

Supplemental data
