# Supplementary material for: Engaging Mortality: Effective Implementation of Dignity Therapy
Source: J Palliat Med. 2024 Jan 30;27(2):176–84. doi: 10.1089/jpm.2023.0336 (PMC10825264; doi:10.1089/jpm.2023.0336)
Supplement: Supplemental data [file Suppl_TableS1.docx]

Supplementary Online Tables S1 to S4

| **Table S1. Examples of the Dignity Therapy Question Protocol*(1)*** |
| --- |
| 1. Tell me about your life history; particularly the parts that you either remember most or think are the most important? When did you feel most alive? |
| 2. Are there specific things that you would want your family to know about you, and are there particular things you would want them to remember? |
| 3. What are the most important roles you have played in life (family roles, vocational roles, community service roles, etc.)? Why were they so important to you, and what do you think you accomplished in those roles? |
| 4. What are your most important accomplishments, and what do you feel most proud of? |
| 5. What are your hopes and dreams for your loved ones? |
| 6. What have you learned about life that you would want to pass along to others? |
| 7. What advice or words of guidance would you wish to pass along to your [son, daughter, husband, wife, parents, other(s)]? |
| 8. Are there particular things that you feel still need to be said to your loved ones, or things that you would want to take the time to say once again? |
| 9. Are there words or perhaps even instructions you would like to offer your family, in order to provide help to prepare them for the future? |
| 10. In creating this permanent record, are their other things that you would like included? |
