# Supplementary material for: Engaging Mortality: Effective Implementation of Dignity Therapy
Source: J Palliat Med. 2024 Jan 30;27(2):176–84. doi: 10.1089/jpm.2023.0336 (PMC10825264; doi:10.1089/jpm.2023.0336)
Supplement: Supplemental data [file Suppl_TableS2.docx]

| **Table S2. Dignity Therapy Intervention Ingredients*(1)*** | | | | |
| --- | --- | --- | --- | --- |
| **Session** | **Timing** | **Purpose** | **Key Ingredients (Features)** | **Process Considerations/Issues** |
| **First**  Nurse-Led or Chaplain-Led DT Contact (Information session) | Visit 1: | To establish relationship with patient.  To explain DT history and procedures. | DT is based on piloted studies.  Sessions are tape-recorded, transcribed, edited, and returned to the patient for feedback.  Process is iterative.  Purpose is a legacy generating document for family or friends.  DT can be free form, guided, or both.  Guide questions may be provided prior to the second meeting upon request.  Recording session is scheduled. | Rapport must be established with patient.  Patient must understand the process.  Nurse/chaplain should be knowledgeable of the process.  Nurse/chaplain should have guide questions available for patient. |
| **Second**  Nurse-led or Chaplain-Led DT Contact (Recording session) | Visit 2:  + 2 weeks | To provide DT.  To record DT session. | Tape-recorded DT session begins with either patient directed content or guide questions.  Session takes about 60 minutes and is highly flexible, accommodating the patient’s desired discussion content.  Nurse/chaplain takes an active role, forming a therapeutic alliance while delivering and organizing the structured intervention.  Legacy document session is scheduled. | Nurse/chaplain must maintain respect, empathy, support, and dignity.  Nurse/chaplain must guide without providing judgment statements.  Tape recorder should be tested prior to session. |
| Intermission (No contact) | Non-visit:  2-4 weeks | To transcribe the session.  To edit the manuscript.  To revise the manuscript.  To produce a legacy document. | Recording session is transcribed by a professional transcriptionist.  Three copies are kept: a) unedited complete transcript, b) ‘tracked’ version of the edited transcript, and c) final edited version.  Single editor initially edits the manuscript: cleaning up the colloquialisms and non-starter stories, adjusting the chronology, and removing stories that may be hurtful or harmful.  Nurse/chaplain reviews the document, making changes with the editor.  Final edited manuscript will end with a summary phrase driven by the patient’s story. | Nurse/chaplain read transcription copy for accuracy prior to editing.  Editor must remain unbiased while editing, making sure the themes come through without changing the content.  Editor must choose an ending to summarize the patient’s story without biasing content.  Timeliness is important. |
| **Third**  Nurse-Led or Chaplain-Led DT Contact (Legacy document session) | Visit 3:  +4 weeks | To deliver edited legacy document.  To receive feedback from patient. | Nurse/chaplain delivers final edited legacy document to the patient.  Nurse/chaplain reads it to the patient and/or the patient will read it alone.  Patient may request editorial changes which will be completed within 24 hours.  If revisions are necessary, nurse/chaplain plans for the final delivery of the legacy document within 24 hours.  Patient plans to deliver the legacy document to his/her family/friends. | Editing may not satisfy the patient.  Theme may not be approved by patient.  Patient may not be able to provide feedback. |
| Final Editing  (if necessary) | Non-visit:  24 hours post Visit 3 | To make final revisions to legacy document. | Nurse/chaplain makes final revisions based on patient feedback and delivers the final legacy document to the patient. | Final revisions are not approved by patient (process closure). |
