# Supplementary material for: Engaging Mortality: Effective Implementation of Dignity Therapy
Source: J Palliat Med. 2024 Jan 30;27(2):176–84. doi: 10.1089/jpm.2023.0336 (PMC10825264; doi:10.1089/jpm.2023.0336)
Supplement: Supplemental data [file Suppl_TableS3.docx]

| Table S3. Dignity Therapy: Exemplars of Repertoire (Perspectives and Practices) Facilitated by Interview and Document Preparation Process***(1)*** | |
| --- | --- |
| Dignity Conserving Repertoire | Ways of looking at one’s situation, or personal actions that can bolster or reinforce a sense of dignity. |
| Dignity Conserving Perspectives | Internally held qualities, often based on long standing personal characteristics, attributes, or world view. |
| Continuity of Self | A sense that the essence of who one is continues to remain intact, in spite of one’s advancing illness. |
| Role Preservation | Ability to continue to function in usual roles to maintain a sense congruence with prior views of self. |
| Generativity/Legacy | The solace and comfort in knowing that something lasting will transcend their death. |
| Maintenance of Pride | The ability to maintain a positive sense of self regard or respect. |
| Hopefulness | An ability to see life as enduring, or having sustained meaning or purpose. |
| Autonomy/Control | A sense of control over one’s life circumstances. |
| Acceptance | The internal process of resigning one’s self to changing life circumstances. |
| Resilience or Fighting Spirit | Mental determination to overcome illness-related concerns and optimize quality of life. |
| Dignity Conserving Practices | Variety of personal approaches/techniques that patients use to bolster or maintain their sense of dignity. |
| Living in the moment | Focusing on immediate issues in the service of not worrying about the future. |
| Maintaining normalcy | Continuous or routine behaviors, which help individuals manage day-to-day challenges. |
| Seeking spiritual comfort | Turning toward or finding solace in one’s religious or spiritual belief system. |
