# Supplementary material for: Engaging Mortality: Effective Implementation of Dignity Therapy
Source: J Palliat Med. 2024 Jan 30;27(2):176–84. doi: 10.1089/jpm.2023.0336 (PMC10825264; doi:10.1089/jpm.2023.0336)
Supplement: Supplemental data [file Suppl_TableS4.docx]

Table S4. Dignity Impact Scale^16^

| Please select the amount you agree with each of the following statements. | | | | | |
| --- | --- | --- | --- | --- | --- |
|  | Strongly disagree | Disagree | Neither agree nor disagree | Agree | Strongly agree |
| The care I received during the past month has given me a sense of looking after unfinished business. | 1 | 2 | 3 | 4 | 5 |
| The care I received during the past month has increased my sense of dignity. | 1 | 2 | 3 | 4 | 5 |
| The care I received during the past month will be of help to my family. | 1 | 2 | 3 | 4 | 5 |
| The care I received during the past month has or could change the way my family sees or appreciates me. | 1 | 2 | 3 | 4 | 5 |
| The care I received during the past month has lessened my sense of sadness and depression. | 1 | 2 | 3 | 4 | 5 |
| The care I received during the past month has made me feel that life was more meaningful. | 1 | 2 | 3 | 4 | 5 |
| The care I received during the past month has given me a heightened sense of purpose. | 1 | 2 | 3 | 4 | 5 |
